# A model to design financially sustainable algorithm-enabled remote patient monitoring for pediatric type 1 diabetes care

**DOI:** 10.3389/fendo.2022.1021982

**Published:** 2022-11-11

**Authors:** Paul Dupenloup, Ryan Leonard Pei, Annie Chang, Michael Z. Gao, Priya Prahalad, Ramesh Johari, Kevin Schulman, Ananta Addala, Dessi P. Zaharieva, David M. Maahs, David Scheinker

**Affiliations:** ^1^ Department of Management Science and Engineering, Stanford University, Stanford, CA, United States; ^2^ Department of Pediatrics, Division of Pediatric Endocrinology, Stanford University, Stanford, CA, United States; ^3^ Stanford Diabetes Research Center, Stanford University, Stanford, CA, United States; ^4^ Clinical Excellence Research Center, Stanford University, Stanford, CA, United States; ^5^ Graduate School of Business, Stanford University, Stanford, CA, United States; ^6^ Department of Medicine, Division of Biomedical Informatics Research, Stanford University, Stanford, CA, United States

**Keywords:** type 1 diabetes (T1D), continuous glucose monitoring (CGM), remote patient monitoring (RPM), algorithm-enabled telemedicine, hemoglobin A1c (HbA1c), pediatrics, health economics, population health

## Abstract

**Introduction:**

Population-level algorithm-enabled remote patient monitoring (RPM) based on continuous glucose monitor (CGM) data review has been shown to improve clinical outcomes in diabetes patients, especially children. However, existing reimbursement models are geared towards the direct provision of clinic care, not population health management. We developed a financial model to assist pediatric type 1 diabetes (T1D) clinics design financially sustainable RPM programs based on algorithm-enabled review of CGM data.

**Methods:**

Data were gathered from a weekly RPM program for 302 pediatric patients with T1D at Lucile Packard Children’s Hospital. We created a customizable financial model to calculate the yearly marginal costs and revenues of providing diabetes education. We consider a baseline or status quo scenario and compare it to two different care delivery scenarios, in which routine appointments are supplemented with algorithm-enabled, flexible, message-based contacts delivered according to patient need. We use the model to estimate the minimum reimbursement rate needed for telemedicine contacts to maintain revenue-neutrality and not suffer an adverse impact to the bottom line.

**Results:**

The financial model estimates that in both scenarios, an average reimbursement rate of roughly $10.00 USD per telehealth interaction would be sufficient to maintain revenue-neutrality. Algorithm-enabled RPM could potentially be billed for using existing RPM CPT codes and lead to margin expansion.

**Conclusion:**

We designed a model which evaluates the financial impact of adopting algorithm-enabled RPM in a pediatric endocrinology clinic serving T1D patients. This model establishes a clear threshold reimbursement value for maintaining revenue-neutrality, as well as an estimate of potential RPM reimbursement revenue which could be billed for. It may serve as a useful financial-planning tool for a pediatric T1D clinic seeking to leverage algorithm-enabled RPM to provide flexible, more timely interventions to its patients.

## Background and aims

The use of continuous glucose monitoring (CGM) is recommended as standard of care by the American Diabetes Association (ADA) for individuals with type 1 diabetes (T1D) and is associated with improved glycemic outcomes and quality of life (1–3). Analyzing and interpreting CGM data for a large number of patients in the clinical setting is challenging in part because of the complexities of data aggregation and a lack of Electronic Medical Record (EMR) integration. In a previous analysis (4–6), we examined a new, telemedicine-based T1D care model based on the use of a remote patient monitoring (RPM) tool that analyzes CGM data and identifies patients likely to benefit from contact from the diabetes care team. This algorithm-enabled tool, known as Timely Interventions for Diabetes Excellence (TIDE), facilitates personalized care for the entire population cared for by the clinic as part of the Teamwork, Targets, Technology, and Tight Control (4T) Study, in which CGM was initiated in the first month for youth with new-onset T1D. In this context, the program – combined with the use of TIDE – was associated with a 0.5% reduction in hemoglobin A1c (HbA1c) and an 86% reduction (4–10) in provider review time per patient. This algorithm-enabled tool, which drastically reduces the per-patient time required for review and ranks patients by who may benefit most from review, may help patients achieve better glycemia while also increasing a clinic’s per-patient capacity. Moreover, algorithm-enabled CGM data review presents an opportunity to expand access to a larger population base, other clinics, and underserved populations, especially those in rural areas which do not have local access to pediatric endocrinologists.

Currently, TIDE is deployed in the context of the 4T Study, meaning that the process of CGM data review is not being billed for. An additional focus of the 4T Study is articulating a sustainable payment model which would allow remote patient monitoring, facilitated by a tool such as TIDE, to be billed for as a component of routine patient care. While the technology is promising (11–13), current provider payment models are based on the direct provision of clinical care, not population health management. Developing robust analytics to serve a T1D population requires investment in data collection, curation, and analysis, and benefits from scale in terms of the amount of data available to clinical staff. While there is recognition that investment is required to develop technology to support high-performing clinical services and Medicare has reimbursement models for RPM, there is not yet an analogous reimbursement model in place for T1D with RPM. Ironically, the benefits of using algorithm-enabled RPM may be the very same factors which also preclude it from being financially sustainable in a traditional Fee-For-Service (FFS) setting. For example, the fact that algorithm-enabled RPM requires significantly less provider time per patient may result in unsustainably low rates of reimbursement or fail to meet the minimum threshold for any reimbursement.

We created a model to design financially sustainable population-level algorithm-enabled RPM based on CGM data review for a diabetes clinic seeking to serve pediatric T1D patients under a hybrid care model where routine appointments delivered by Certified Diabetes Care and Education Specialists (CDCES) are supplemented with flexible, timelier message-based contacts delivered according to patient need. Though these messages are not like-for-like replacements for in-depth education, they can provide quick, actionable feedback to “nudge” patients in a positive direction (14). This model explores a capacity-neutral scenario in which a clinic incorporates algorithm-enabled telemedicine into T1D care and a scenario requiring additional capacity. While maintaining clinic capacity, we examine the feasibility of decreasing the frequency of routine diabetes education visits and repurposing the existing capacity for algorithm-enabled telemedicine. When augmenting clinic capacity, we consider the feasibility of maintaining the regular cadence of routine visits and delivering algorithm-enabled telemedicine services as a supplement to routine care. In both scenarios, our aim is to quantify the financial impact of transitioning to algorithm-enabled telemedicine on a clinic’s bottom line and estimate the minimum reimbursement rate necessary from telemedicine interactions to maintain cost-neutrality while reproducing previously observed improvements to A1c.

The one-time fixed costs of developing and deploying the necessary hardware and software vary significantly. For institutions with an established telemedicine program, the costs would primarily stem from the provider time for operational planning, training, and patient recruitment. These costs also apply to institutions interested in using free, online, open-source tools, as well as those with Tableau Server already deployed interested in using the free Tableau-based tool. For institutions interested in deploying with enterprise data visualization software (e.g., Tableau or PowerBI) with automatic feeds from the electronic medical record, costs can range from tens of thousands to hundreds of thousands of dollars per year (depending on the server type and the number of users). Of course, most institutions would likely use such enterprise software for a variety of projects and initiatives. For a full integration with the electronic medical record, the costs are difficult to estimate, due to the difficulty of working with modern EMRs, but could be on the order of hundreds of thousands of dollars and require months of effort. In this work we focus primarily on the marginal costs of deploying an algorithm-enabled tool, since an estimate of these costs would be a central component in deciding how much to invest in launching the program.

Although this analysis is grounded in our experience with TIDE in the context of the 4T research study at Lucile Packard Children’s Hospital Stanford (LPCH), it is broadly applicable to any clinic currently using their own version of a tool like TIDE, or seeking to build an algorithm-enabled tool which allocates capacity based on patient need. Moreover, though this analysis primarily applies to the US medical system, concepts of scalability are generalizable to other health care systems.

## Methods

### Setting and population

The protocol for the 4T Program (8), as well as a summary timeline showcasing the major milestones of the program (15), have been previously described. Briefly, all youth with newly diagnosed T1D between July 2018 and June 2020 were offered the opportunity to start on CGM (Dexcom G6, Dexcom Inc., San Diego, CA) in the first month of diabetes diagnosis following the clinic’s standard of care. Those who chose to start on CGM had a follow-up visit (either in-person or telemedicine) with a CDCES to initiate CGM. At this visit, participants were provided with CGM supplies (i.e., a transmitter, three sensors, and a receiver) by the 4T Study team. The diabetes care team applied for ongoing insurance approval for CGM coverage and if it was not covered by insurance, the research study provided CGM supplies. One week after initiating CGM, the youth and family were encouraged to meet with a nurse practitioner *via* telemedicine for a follow-up visit for additional CGM education and continued with routine care.

Youth diagnosed in March 2019 or later were additionally offered the opportunity to participate in remote patient monitoring (clinical trial No. NCT03968055). Data were shared from the patient’s device to the Dexcom Clarity cloud-based platform. To facilitate CGM data sharing, youth who did not have their own iOS device were provided with an iPod touch (Apple Inc., Cupertino, CA) for the duration of the study. Each week, CGM data were reviewed by a CDCES and when necessary, insulin dose adjustments were recommended using secure messaging within the EMR system. Initially, CDCES manually reviewed the CGM glucose data of every participant, but by January 2020, they relied on the TIDE population health management tool to facilitate 4T Study scaling to a larger patient population (4–6). The health management tool was based on CGM consensus guidelines for percent time in range (TIR; 70-180 mg/dL), time in hypoglycemia (<70 mg/dL), and time in clinically significant hypoglycemia (<54 mg/dL) (16). The modified tool prioritized patients for CDCES review by identifying participants with TIR of less than 65%, or percentage of time CGM was worn less than 50% over a one week period to increase scalability with constrained CDCES time, but the flags for hypoglycemia were unchanged (8). This protocol was approved by the Stanford Institutional Review Board (IRB) and informed consent (and assent for participants aged 7-18 years) was obtained for all participants.

### Study population

TIDE is currently in use for youth in four institutional review board–approved studies: 4T pilot, 4T phase 1, 4T phase 2 (15), and CGM Time in Range Program at Stanford (CGM TIPS). Among the four, TIDE is now used to support scaling RPM to 299 youth with T1D (**Table 1**). All of those enrolled gave informed consent for the care team to review the data collected by their CGM every week and to send them a message with suggestions for glucose management, when appropriate.

**Table 1:** Participants demographics for the 4T Pilot Study, 4T Study 1, 4T Study 2, and CGM TIPS Study.

	Study			
	Pilot	4T Study 1	4T Study 2	CGM TIPs
**Participants, N**	135	133	31	94
**Age at T1D diagnosis, median (Q1-Q3), y**	9.7 (6.8-12.7)	11 (7–14)	11 (9.5-14.0)	9.0 (5.2 - 11.6)
**Sex**				
Female, n (%)	64 (47.4)	60 (45)	20 (64.5)	48 (51.1)
Male, n (%)	71 (52.6)	73 (55)	11 (35.5)	46 (48.9)
**Race/Ethnicity, n (%)**				
Non-Hispanic White	50 (37.0)	43 (32.3)	7 (22.6)	18 (19.1)
Non-Hispanic Black	0 (0)	0 (0)	0 (0)	5 (5.3)
Hispanic	25 (18.5)	38 (28.6)	3 (9.7%)	40 (42.6)
Asian or Pacific Islander	17 (12.6)	13 (9.8)	3 (9.7%)	2 (2.1)
American Indian or Alaska Native	0 (0)	0 (0)	0 (0)	0 (0)
Other	11 (8.1)	13 (9.8)	12 (38.7)	7 (7.4)
Unknown	32 (23.7)	26 (19.5)	6 (19.4)	22 (23.4)
**Insurance Type, n (%)**				
Private	104 (77.0)	80 (60.2)	28 (90.3)	6 (6.4)
Public	31 (23.0)	47 (35.3)	3 (9.7)	82 (87.2)
Both	0 (0)	2 (1.5)	0 (0)	4 (4.3)
Unknown or No Insurance	0 (0)	4 (3.0)	0 (0)	2 (2.1)

### Overview

We created a financial model to calculate the yearly marginal costs and revenues of providing diabetes education from the point of view of a diabetes clinic serving pediatric T1D patients. We evaluated two different care delivery scenarios and compared them to a status quo baseline scenario.

In the baseline scenario, diabetes education is provided by CDCES at a fixed, regular cadence and reimbursed under a traditional FFS model. The incentives under this system are to ensure the maximum utilization of each resource, resulting in scheduled appointments with no allocation to flexible capacity. In this model, services are not optimized for patients requiring more assistance. Rather, all patients receive the same number of appointments, which are regularly scheduled throughout the year.

**Figure 1:**
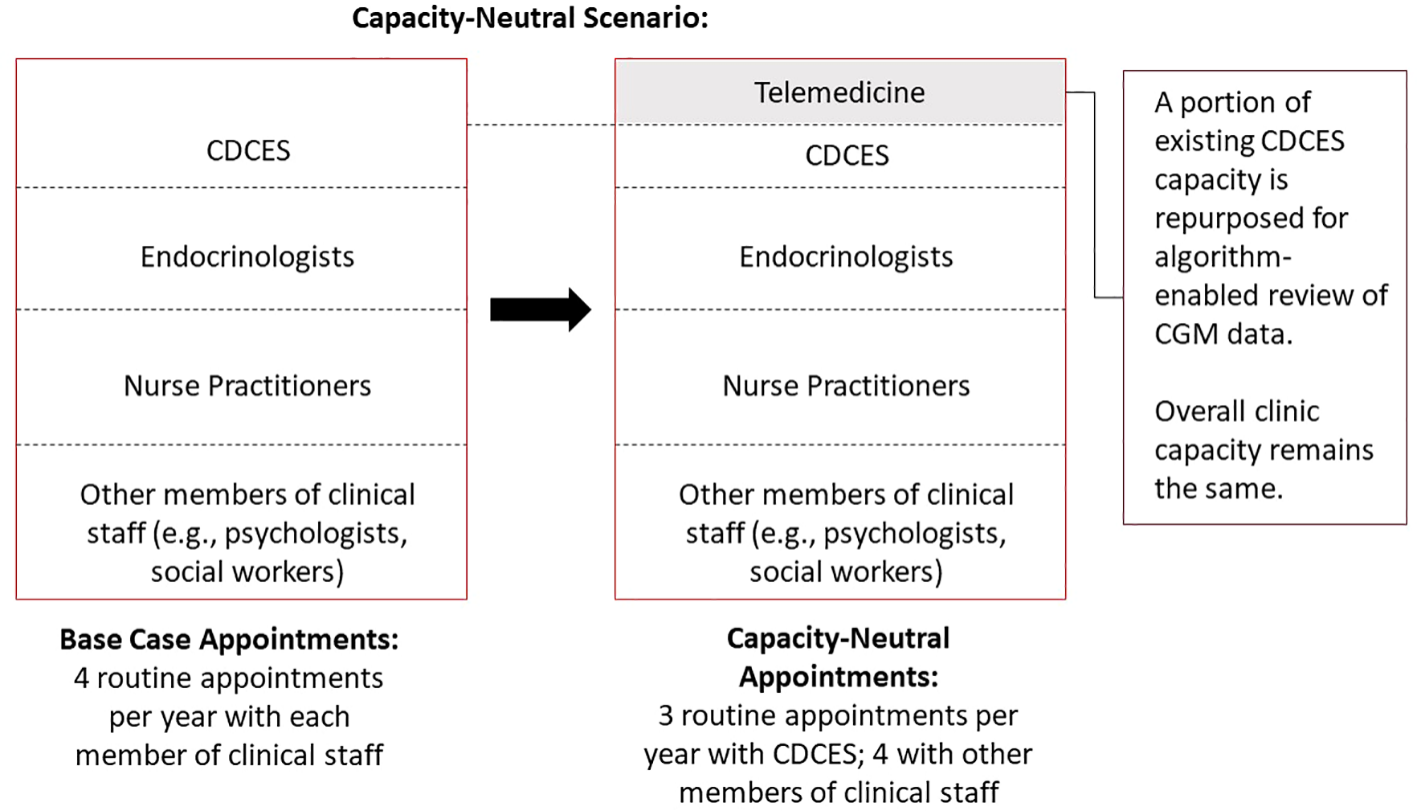
Schematic diagram of capacity-neutral scenario, whereby a clinic decreases the frequency of routine diabetes education visits and repurposes the existing capacity for algorithm-enabled telemedicine.

In a capacity-neutral scenario, we assumed that the clinic shifted to population-level algorithm-enabled RPM based on CGM data review. Some proportion of existing CDCES capacity allocated for regularly scheduled visits is re-allocated to flexible telemedicine visits reserved for patients who are most likely to benefit from contact. Rather than augmenting the clinic capacity with additional resources, this telemedicine capacity is drawn from existing resources, and the capacity devoted to routine care is reduced accordingly. As a result, the overall effect on clinic capacity is neutral, as is the effect on clinic costs (**Figure 1**).

**Figure 2:**
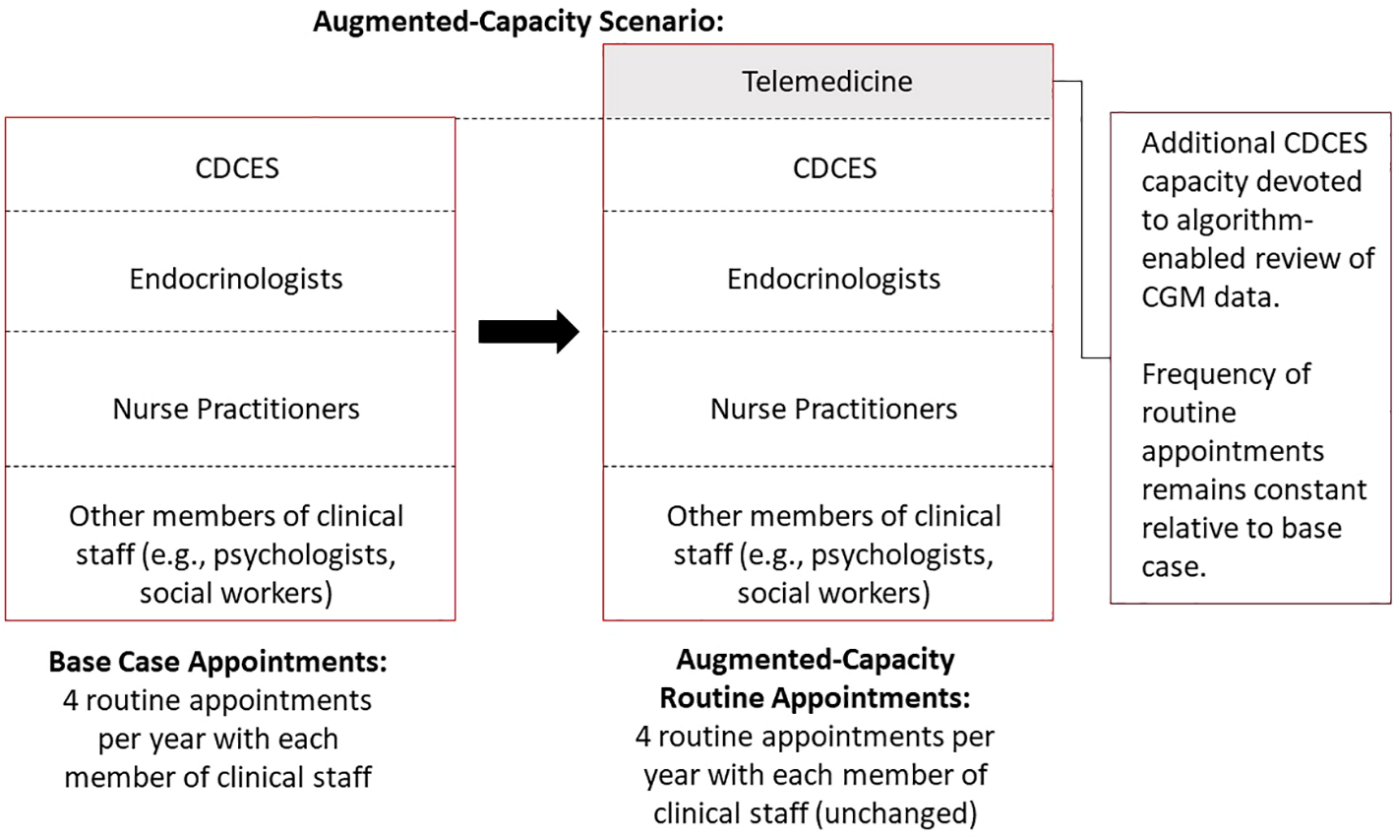
Schematic diagram of augmented-capacity scenario, whereby a clinic maintains all routine appointments but increases its overall capacity to deliver supplemental, algorithm-enabled telemedicine interactions.

In an augmented-capacity scenario, we assumed that the clinic maintained all routine appointments, but increased its overall capacity to deliver algorithm-enabled telemedicine over and above its existing care model. Moreover, the clinic incurs additional marginal labor costs to meet the increased CDCES capacity requirements (**Figure 2**).

In both population health scenarios, telemedicine capacity is flexible and allocated to patients with the highest need in each time period (4–6). In both cases, we then estimated the minimum reimbursement rate needed for telemedicine contacts to offset this revenue gap. If this payment threshold is met, the effect on the overall clinic bottom line is neutral. In addition, we evaluated the marginal revenue which could potentially be billed for using RPM billing codes and compared this to the break-even threshold value.

### Base case

We modelled a cohort of 100 pediatric patients served by a T1D clinic. We assumed that patients received one diabetes education session every quarter (four visits per year), which is scheduled alongside concurrent routine appointments with other members of the clinical staff (e.g., endocrinologists, registered dieticians, psychologists). We assumed that diabetes education was provided entirely by the CDCES team at a fixed, regular cadence. We assumed an average per appointment reimbursement rate of $56.00 USD, which is reflective of the average reimbursement rate as per the 2022 Medicare National Fee Schedule for Diabetes outpatient self-management training services delivered by a CDCDS in an ADA-recognized program (per individual, per 30 minutes - CPT code G0108)^1^. This reimbursement rate is varied in sensitivity analysis and can be modified to reflect differences in state-specific rates, as well as payer environment and composition (e.g., to account for a mix of private and public insurance plans with differential rates of reimbursement). Our model estimates the total marginal yearly reimbursement revenue from patients receiving diabetes education sessions at this fixed cadence. Given that our model only considers modifications to CDCES capacity and appointments, we did not estimate marginal revenue stemming from appointments with other members of the clinical staff, as these remain constant throughout our modeling scenarios.

We assumed that on average, a CDCES conducts five education sessions per day, five days per week, for a total of 25 education sessions per week. Over the course of 52 weeks per year, this corresponds to a total yearly capacity of 1,300 education sessions. Using this estimate of capacity, we determined the number of full-time equivalent hours (FTE) necessary to provide coverage for our patient population. By pairing this with the average US national yearly salary of a CDCES reported by a commercial compensation database^2^, we then calculated the total marginal CDCES labor costs associated with delivering care to the clinic population (**Table 2**). These base case parameters are defined as modifiable in our model.

**Table 2:** Key input parameters for base case under status quo, in which diabetes education is provided by CDCES at a fixed, regular cadence and reimbursed under a traditional FFS model.

Base Case	Value
Number of patients in practice	100
Routine visits per patient per year	4
Reimbursement rate per routine visit	$56
**Marginal Reimbursement Revenue**	$22,400
Annual CDCES salary	$85,000
CDCES capacity (appointments per year)	1,300
Total appointments needed (per year)	400
FTE needed	0.31
**Total CDCES labor costs**	$26,154

### Capacity-neutral scenario

In the capacity-neutral scenario, our assumptions for the size of the patient population, the reimbursement rate, and the CDCES labor costs and capacity remain constant relative to the base case scenario. However, we assumed that some of the existing CDCES capacity reserved for routine appointments is reallocated for flexible telemedicine contacts (i.e., for reviewing patients identified by algorithm-enabled CGM data review, and – if necessary – sending them a message through the EMR to suggest a dose change (4–6)). We assumed that one out of the four quarterly routine CDCES appointments was repurposed for telemedicine, meaning that all patients received one less routine education session per year (other appointments with other members of the clinical staff remain unchanged). However, given that algorithm-enabled data review takes up significantly less provider time than a routine diabetes education appointment, repurposing routine appointments frees up capacity for multiple flexible, telemedicine contacts. Assuming that an average routine appointment lasts 30 minutes, compared to 5 minutes for a message-based contact, then repurposing one routine appointment per patient frees up sufficient capacity for up to six message-based contacts.

Given a population of 100 patients, this flexible telemedicine capacity amounts to a total of 600 message-based contacts per year, or 50 per month, which provides telemedicine coverage for 50% of the total patient population in every monthly review period. We assumed that all of this capacity is utilized by the 50% of patients who meet the criteria for intervention. Note that this capacity is flexible, and not earmarked for any patients in particular. Given that algorithm-enabled RPM ranks and identifies patients who are most likely to benefit from contact, some patients may receive more contacts than others in a given year, while some may not receive any.

We then calculated the marginal revenue lost from repurposing routine education sessions. Based on the number of message-based contacts delivered, we estimated the minimum reimbursement rate per telemedicine contact necessary to offset this revenue loss and maintain strict revenue-neutrality. In addition, we calculated the potential marginal revenue which could be billed for using existing RPM CPT codes and compared this to the break-even threshold value. We assumed an average reimbursement rate of $35.00 USD per telemedicine-based contact, which reflects the average reimbursement rate as per the 2022 Medicare National Fee Schedule for analysis and interpretation of CGM data (CPT code 95251)^3^ (**Table 3**).

**Table 3:** Key input parameters for capacity-neutral scenario, in which a proportion of existing CDCES capacity for routine appointments is repurposed for dynamically allocated, algorithm-enabled contacts.

Capacity-neutral scenario	Value
In person visits per patient per year	3
Marginal Reimbursement Revenue	$ 16,800
**Revenue Gap**	$ 5,600
Appointments repurposed for telehealth	100
Duration of in-person visit (mins)	30
Duration of telemedicine appointment (mins)	5
Total telemedicine appointments freed up	600
**Minimum reimbursement rate per telemedicine appointment**	$9.33
RPM reimbursement rate	$35
Potential marginal RPM reimbursement revenue	$ 21,000
**Margin**	$ 15,400

### Augmented-capacity scenario

In the augmented-capacity scenario, our assumptions for the size of the patient population, reimbursement rate, and the frequency of routine visits remained constant relative to the base case. However, we assumed that the clinic increased its CDCES capacity in a two-step approach. In the first year, we assumed a capacity increase sufficient to review data for up to 75% of patients in any given review period (which is in line with the proportion of patients failing to meet the target HbA1c observed in our baseline population and the US pediatric T1D population) (9, 17). We assumed that more intensive management of patients who did not reach HbA1c targets in the first year improved overall outcomes on the long-term, and that in the second year, the clinic would reduce this extra capacity to provide algorithm-enabled telemedicine coverage for 50% of its patient population. This mirrors the improved, long-term outcomes observed over the course of the 4T Study (9). Moreover, these parameters are modifiable, and can be adjusted to reflect the makeup of a clinic’s patient population.

Given a population of 100 patients, these additional capacity requirements translate to 75 telemedicine interactions needed per month in the first year (i.e. 900 total), and 50 needed per month in the second year (i.e. 600 total). Based on the yearly capacity of a CDCES, we estimated the additional FTE needed to deliver these appointments. We then calculated the additional marginal labor costs incurred from increasing CDCES capacity. Based on the number of message-based contacts delivered, we then estimated the minimum telemedicine reimbursement rate per telemedicine contact necessary to offset these additional marginal costs and maintain strict revenue-neutrality. In addition, we calculated the potential marginal revenue which could be billed for using existing RPM CPT codes, assuming an average reimbursement rate of $35.00 USD per telemedicine-based contact as in the capacity-neutral scenario^4^ (**Table 4**).

**Table 4:** Key input parameters for augmented-capacity scenario, in which clinic maintains all routine appointments, but increases overall capacity to deliver algorithm-enabled telemedicine over and above its existing care model.

Augmented-Capacity Scenario	Value
**Year 1**	
Percentage of patients receiving telemedicine	75%
Telemedicine appointments needed per year	900
CDCES capacity (appointments per year)	1300
Additional FTE needed	0.12
Additional marginal CDCES labor costs	$ 9,808
**Minimum reimbursement rate per telemedicine appointment**	$10.90
RPM reimbursement rate	$35
Potential marginal RPM reimbursement revenue	$ 31,500
**Margin**	$ 21,692
**Year 2**	
Percentage of patients receiving telehealth	50%
Telehealth appointments needed	600
CDCES capacity (appointments per year)	1300
Additional FTE needed	0.08
Additional marginal CDCES labor costs	$ 6,538
**Minimum reimbursement rate per telemedicine appointment**	$10.90
RPM reimbursement rate	$35
Potential marginal RPM reimbursement revenue	$ 21,000
**Margin**	$ 14,462

The input parameters for both scenarios are comparable to the ones observed in our patient population and are defined as modifiable in our financial model.

### Projecting impact of improved outcomes

Given that the 4T program and the use of the TIDE tool to identify participants who would benefit from dose adjustments was associated with a 0.5% reduction in HbA1c (9), we examine scenarios in which personalized, timely, telemedicine-based interventions may lead to long-term improved patient outcomes (18), and therefore reduced healthcare costs in the long-term (e.g., by reducing the incidence of diabetic ketoacidosis and chronic vascular complications, or by driving down the demand for CGM data review). Accordingly, as an additional input for the model, a user may indicate how much a 0.5% improvement in HbA1c would be worth to their clinic, on average per patient. The value stemming from improved patient outcomes is then subtracted from the revenue gap to calculate the resulting, reduced minimum reimbursement rate to ensure strict revenue-neutrality.

### Sensitivity analysis

We varied the number of routine appointments delivered per year, the corresponding reimbursement rate, the CDCES capacity, the CDCES salary, the number of routine appointments shifted to telemedicine, the average time per appointment, and the value of a 0.5% improvement in HbA1c in one-way sensitivity analyses, and evaluated the impact on the minimum reimbursement rate.

## Results

### Financial impact of capacity-neutral scenario

**Figure 3:**
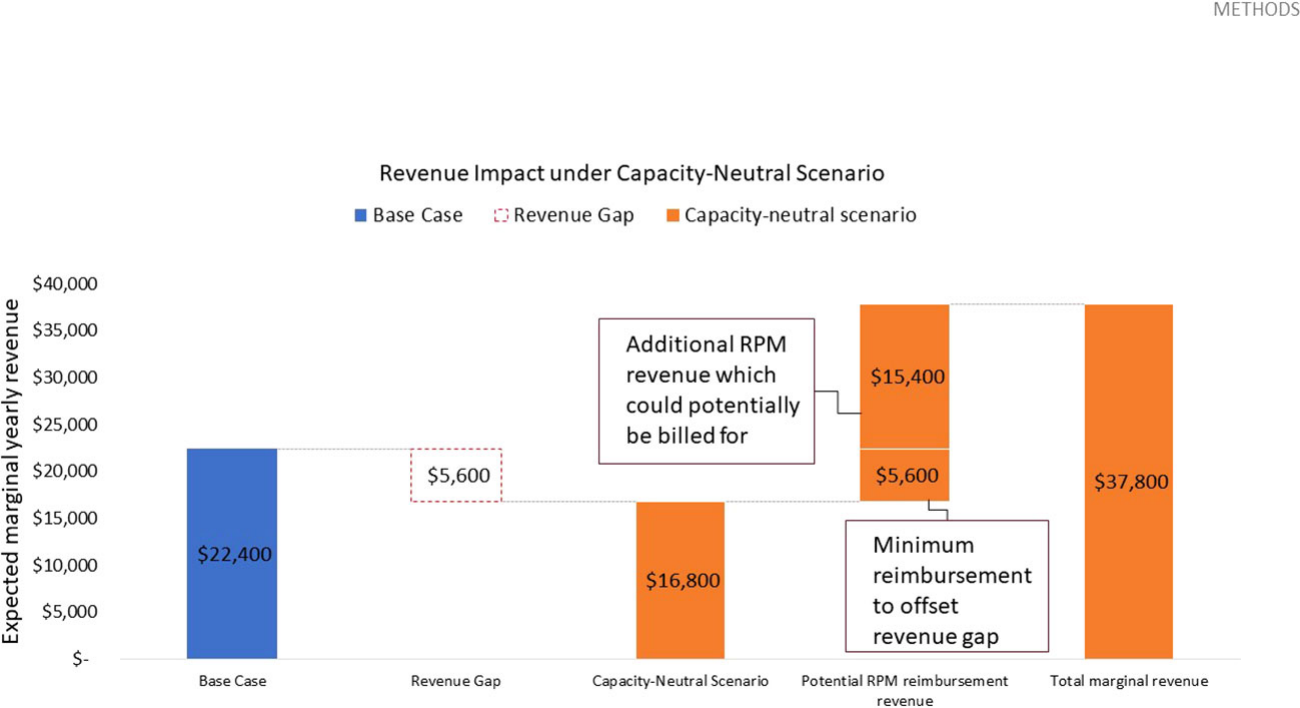
Expected yearly marginal revenue under capacity-neutral scenario, assuming 600 total telehealth appointments delivered, a $9.33 USD reimbursement rate to offset the revenue gap, and a potential RPM reimbursement rate of $35.00 USD based on existing CPT codes.

For an initial cohort of 100 pediatric patients, shifting one out of four yearly routine diabetes education appointments to telemedicine translates to repurposing 100 diabetes education sessions. Given these are reimbursed at a rate of $56.00 USD per session^5^, this results in a marginal revenue loss of $5,600.00 USD per year. As telemedicine interactions only take 5 minutes (vs 30 minutes for an education session), sufficient capacity is freed up for 600 messaged-based contacts per year. Assuming these are flexibly booked out in their entirety in each review period, these telemedicine interactions would need to be reimbursed at a minimum rate of roughly $9.33 USD per contact to bridge the revenue gap. In this capacity-neutral view, no additional investment in clinic resources would be necessary, and CDCES labor costs remain constant. Given that remote CGM analysis and interpretation is currently reimbursed at a rate of $35.00 USD^6^, the potential marginal RPM revenue which could be billed for is sufficient to offset this revenue loss. In fact, this marginal RPM revenue totals $21,000.00 USD and results in a margin increase of $15,400.00 USD, relative to the base case (**Table 3** and **Figure 3**).

### Financial impact of augmented-capacity scenario

For an initial cohort of 100 pediatric patients, adding sufficient telemedicine capacity to cover 75% of patients in each review period translates to 900 telemedicine interactions in year 1. Given that a CDCES, on average, can conduct 1,300 education sessions per year – or 7,800 telemedicine contacts per year – this translates to an additional 0.12 FTE. Given a yearly salary of $85,000.00 USD for a CDCES^7^, the clinic incurs an additional $9,808.00 USD in marginal CDCES labor costs in year 1. In year 2, assuming sufficient telemedicine capacity to provide coverage for 50% of patients (which translates to 600 telemedicine interactions), the clinic requires an additional 0.08 FTE and incurs an additional $6,538.00 USD in marginal labor costs. If flexibly booked out in their entirety in each review period, these telemedicine interactions would need to be reimbursed at a minimum rate of roughly $10.90 USD per contact to bridge the revenue gap, in both year 1 and year 2. In this augmented-capacity view, the frequency of routine appointments remains unchanged, and the corresponding marginal revenue remains constant.

**Figure 4:**
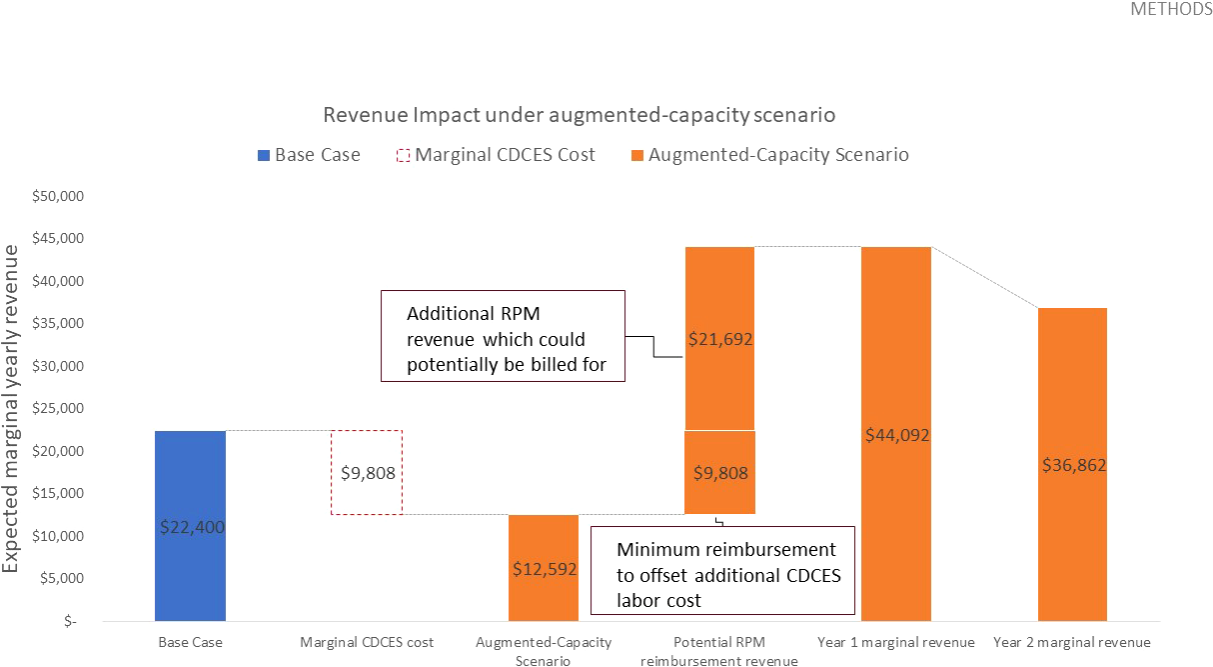
Expected yearly marginal revenue under augmented-capacity scenario, assuming 900 total telehealth appointments delivered in year 1 and 600 in year 2, a $10.90 USD reimbursement rate to offset the additional CDCES labor costs, and a potential RPM reimbursement rate of $35.00 USD based on existing CPT codes.

Given that remote CGM analysis and interpretation is currently reimbursed at a rate of $35.00 USD^8^, the potential marginal RPM revenue which could be billed for is sufficient to offset this revenue loss. In fact, in year 1, this marginal RPM revenue totals $31,500.00 USD and results in a margin increase of $21,692.00 USD, relative to the base case. In year 2, this marginal RPM revenue totals $21,000.00 USD and results in a margin increase of $14,462.00 USD, relative to the base case (**Table 4** and **Figure 4**).

A side-by-side summary of the key inputs and outputs of the model for the base case, capacity-neutral, and augmented-capacity scenarios is included in **Table 5**.

**Table 5:** Side-by-side comparison of key input and output parameters of the financial model for base case, capacity-neutral scenario, and augmented-capacity scenario.

Parameters	Base case	Capacity-Neutral scenario	Augmented-Capacity Scenario
Routine visits per patient per year	4	3	4
Reimbursement rate per routine visit	$56	$56	$56
CDCES capacity (appointments per year)	1,300	1,300	1,300
Annual CDCES salary	$85,000	$85,000	$85,000
Duration of in-person visit (mins)	30	30	30
Duration of telemedicine appointment (mins)	5	5	5
Routine appointments repurposed for telehealth	N/A	100	0
Lost revenue from routine appointments	N/A	$5,600	0
Additional FTE needed	N/A	0	0.12
Additional marginal CDCES labor costs	N/A	0	$9,808
Minimum reimbursement rate per telemedicine appointment	N/A	$9.33	$10.90

N/A means not applicable for the base case.

### Sensitivity analysis

In the capacity-neutral scenario, the minimum telemedicine reimbursement rate was sensitive only to the routine appointment reimbursement rate, the average time per visit, and the value of a 0.5% improvement in HbA1c. The routine reimbursement rate determines the base case marginal reimbursement revenue received by the clinic, and therefore the magnitude of the revenue loss when transitioning to telemedicine. The average time per visit determines the number of message-based contacts that can be freed up by repurposing a routine appointment, and therefore the fraction of lost marginal revenue that each message-based contact must recoup. If repurposing one routine visit per patient frees up sufficient capacity for six telemedicine visits per patient, then the minimum telemedicine reimbursement rate is one sixth of the routine reimbursement rate to maintain revenue-neutrality (**Table 6**).

**Table 6:** Selected results of one-way sensitivity analyses, showing key input parameters which have an impact on the minimum reimbursement rate per telemedicine appointment needed to maintain revenue-neutrality.

Sensitivity Analysis	Minimum reimbursement rate per telemedicine appointment
**Capacity-Neutral Scenario**	$ 9.33
50% increase in routine appointment reimbursement rate	$ 14.00
50% decrease in routine appointment reimbursement rate	$ 4.67
50% increase in average time per routine appointment	$ 6.22
50% decrease in average time per routine appointment	$ 18.67
**Augmented-Capacity Scenario**	$ 10.90
50% increase in salary of CDCES	$ 16.35
50% decrease in salary of CDCES	$ 5.45
50% increase in capacity of CDCES	$ 7.26
50% decrease in capacity of CDCES	$ 21.79

In the augmented-capacity scenario, the minimum telemedicine reimbursement rate was sensitive only to the salary of a CDCES, the capacity of a CDCES, and the value of a 0.5% improvement in HbA1c. Given that the additional marginal labor costs are proportional to the number of appointments delivered, it stands to reason that the capacity and the salary of a CDCES would have a linear effect on the marginal costs incurred (**Table 6**).

In both cases, as the value derived by the clinic from a 0.5% improvement in HbA1c increases, the magnitude of the revenue gap shrinks. Beyond a certain threshold value, the benefit of achieving improved outcomes may be sufficient in and of itself to recoup the lost reimbursement revenue. In a healthcare system in which patients are cared for long-term, reductions in long-term complications which are predicted by HbA1c may lead to cost savings.

## Discussion

We created a model to design financially sustainable algorithm-enabled CGM data review for a clinic that serves pediatric T1D patients. This model is aligned with the 4T Study and the TIDE tool, which has been deployed for population health management of our patients but has not yet been billed for as a routine component of diabetes care. While this model closely tracks the characteristics of our environment, it has broad applicability beyond our clinic, and can serve as a capacity- and financial-planning tool for a pediatric T1D clinic seeking to leverage algorithm-enabled RPM to provide flexible, more timely interventions to its patients. Given the rapid transition to remote care spurred by the COVID-19 pandemic (19), T1D clinics expanding their telemedicine offerings may be especially interested in population-level algorithm-enabled RPM based on CGM data review, whose unique value proposition includes reduced administrative burden, reduced provider review time per patient, more intensive, targeted management of patients failing to meet HbA1c targets, improved patient and parent satisfaction, and improved patient quality of life and glycemic outcomes. Financial planning can be facilitated by and should be done in complement with operational planning. To that end, this financial model can be used in combination with the capacity planning dashboard developed by our team to contribute to care delivery that is both operationally efficient and financially sustainable (20).

However, T1D clinics may be reticent to invest in deploying this tool out of a concern that it may unduly disrupt clinic operations and lead to increased costs. To address these concerns, we have explored two possible pathways to adopting algorithm-enabled RPM. While some institutions may wish to convert their existing capacity, others may have a financially viable path to making an upfront investment in expanding their CDCES capacity. In both cases, our analysis reveals that despite a substantially lower reimbursement rate for remote analysis and interpretation of CGM data compared to a routine diabetes education session ($35.00 vs $56.00 USD), algorithm-enabled CGM data review is financially sustainable due to its scalability. By expediting data analysis and interpretation and cutting down on provider review time, algorithm-enabled RPM allows monitoring multiple patients in the same time that it would take to provide a diabetes education session to a single patient. As a result, the lower reimbursement rate is offset by the sheer volume of telemedicine-based contacts that can be provided in a comparable timeframe. Moreover, it is important to note that our estimates of potential reimbursement are conservative. For example, we have only considered the potential revenue stemming from CGM interpretation and analysis, however other CPT codes (e.g., 95250 for initial training and set-up of CGM) could also be billed for. Moreover, we have assumed an average reimbursement rate in line with the 2022 Medicare National Fee Schedule. However, in practice reimbursement rates for privately insured patients is typically higher.

Even if this level of RPM reimbursement is not achievable, there are other reasons to believe that the long-term benefits of using algorithm-enabled RPM may justify its initial investment. A population health model of care which dynamically allocates capacity to patients who need it the most is likely to lead to additional sources of long-term cost savings stemming from improved patient outcomes. Moreover, reducing the provider review time per patient increases the number of patients that can be seen per provider, which translates to reduced FTE (and its attendant cost savings), an increased patient population, additional visits per patient beyond the recommended four per year, or some combination of these.

We recognize that there are limitations in our model. Though our algorithm-enabled tool has been made available as open-source software, additional ancillary costs may need to be factored into the cost of deploying the tool (e.g., hosting, technical support, customization costs), and may therefore increase the minimum reimbursement rate. In particular, our model does not account for the one-time fixed costs of developing and deploying the necessary hardware and software, can be used as a financial tool to assist stakeholders in deciding how much to spend on fixed costs. In the capacity-neutral view, we assumed that one out of the four quarterly routine appointments is repurposed for telemedicine, however in practice a clinic may not wish to reduce routine appointments unilaterally for all patients (e.g., new-onset patients typically require additional appointments in the first year after diagnosis). We assumed that all flexible, telemedicine capacity could be fully booked out in any given monthly review period, and that none of this capacity would sit idle. In the most extreme case, our augmented-capacity model assumed that in year 1, 75% of patients would meet criteria for review. Although it is possible that fewer patients would be eligible for a telemedicine-based contact, it is important to note that the ADA HbA1c goal of <58 mmol/mol for youth was achieved by only 17% of youth between 2016 and 2018 (17). In our patient population, nearly half of patients still did not reach HbA1c targets after one year and continued to benefit from support from a 4T treatment model (9). Given that a majority of patients still do not meet HbA1c targets in the US, it would appear reasonable to assume that the entirety of this flexible capacity could be safely booked out in any given review period, and that no slack capacity would go to waste.

Though this analysis is grounded in the characteristics of TIDE and our patient population, the parameters of this financial model can be customized to address the factors specific to another care provider’s clinic, population, and payer environment. However, it is important to note that the 4T model and TIDE tool were used in study populations and may not be generalizable to the wider patient population at this time. Future directions include scaling this beyond new onset T1D and adapting these concepts for adult T1D patients and patients with type 2 diabetes (T2D). Of course, this model is not directly applicable to patients without access to a CGM, which underscores the need for increased coverage in under- or un-insured families who would otherwise not benefit from algorithm-enabled CGM data review.

## Conclusion

We created a model to design financially sustainable CGM-based algorithm-enabled RPM based on data from 4 studies at a single site. Our model establishes a clear threshold reimbursement value for maintaining revenue-neutrality, as well as an estimate of potential marginal RPM reimbursement revenue which could be billed for. The minimum reimbursement rate for telemedicine interactions will ultimately depend on a given clinic’s pathway to adopting algorithm-enabled RPM (i.e., repurposing vs. expanding capacity), as well as the value they derive from improving patient outcomes on the long-term. Existing reimbursement levels for RPM suggest that algorithm-enabled CGM data review may in fact increase marginal revenues for T1D clinics. Our analysis may inform operational and financial planning at the clinic level as well as reimbursement policy design to facilitate the transition to remote diabetes care.

## Author’s note

A complete list of members of the 4T Study Research Team appears in the Acknowledgments.

## Data availability statement

The original contributions presented in the study are included in the article/supplementary material. Further inquiries can be directed to the corresponding author.

## Author contributions

PD performed the research and designed the model. PD prepared the manuscript with input from all authors. AC and MG designed the capacity planning dashboard. DZ, DM, PP, and KS provided clinical guidance and input on the manuscript. RJ and DS supervised the project and provided input on the manuscript. All authors contributed to the article and approved the submitted version.

## Funding

This work was supported by the ISPAD-JDRF Research Fellowship (Grant R18DK122422); the Helmsley Charitable Trust (G-2002-04251- 2); the Stanford Diabetes Research Center (1P30DK11607401); Lucile Packard Children’s Hospital Auxiliaries; the Stanford Maternal and Child Health Research Institute (MCHRI); and the Stanford Institute for Human-Centered Artificial Intelligence (HAI).

## Acknowledgments

We would like to thank the other members of the 4T Study Group for their help with this project: Brianna Leverenz, BS; Julie Hooper MPH, RD; Ana Cortes, BS; Nora Arrizon-Ruiz; Erica Pang, BS; Carolyn Herrera, BS; Rachel Tam; Dom Mitchell, BS; Liz Heckard, BS; Andrea Bonilla Ospina, BS; Franziska Bishop, MS, CDCES; Natalie Pageler, MD; Jeannine Leverenz, RN, CDCES; Piper Sagan, RN, CDCES; Anjoli Martinez-Singh, RD, CDCES; Barry Conrad RD, CDCES; Annette Chmielewski, RD, CDCES; Julie Senaldi RN, CDCES; Ilenia Baroro, BS; Molly Tanenbaum, PhD; Connor Brown, BS; and Glenn Loving, MS.

## Conflict of interest

PP is affiliated with the Stanford Diabetes Research Center. DZ has received speaker’s honoraria from Medtronic Diabetes, Ascensia Diabetes, and Insulet Canada; and research support from the Helmsley Charitable Trust and ISPADJDRF research Fellowship. She is also on the Dexcom Advisory board. DM has had research support from the National Kidney Foundation, Juvenile Diabetes Research Foundation, NSF, and the Helmsley Charitable Trust and his institution has had research support from Medtronic, Dexcom, Insulet, Bigfoot Biomedical, Tandem, and Roche. DM has consulted for Abbott, Aditxt, the Helmsley Charitable Trust, Lifescan, Mannkind, Sanofi, Novo Nordisk, Eli Lilly, Medtronic, Insulet, Dompe, and Biospex. DM is affiliated with the Stanford Diabetes Research Center. DS is advisor to Carta Healthcare.

The remaining authors declare that the research was conducted in the absence of any commercial or financial relationships that could be construed as a potential conflict of interest.

## Publisher’s note

All claims expressed in this article are solely those of the authors and do not necessarily represent those of their affiliated organizations, or those of the publisher, the editors and the reviewers. Any product that may be evaluated in this article, or claim that may be made by its manufacturer, is not guaranteed or endorsed by the publisher.

## Footnotes

1
https://www.cms.gov/medicare/physician-fee-schedule/search?Y=0&T=4&HT=0&CT=0&H1=G0108&M=5

2
https://www.salary.com/research/salary/benchmark/diabetes-educator-salary

3
https://www.cms.gov/medicare/physician-fee-schedule/search?Y=0&T=4&HT=0&CT=0&H1=95251&M=5

4
https://www.cms.gov/medicare/physician-fee-schedule/search?Y=0&T=4&HT=0&CT=0&H1=95251&M=5

5
https://www.cms.gov/medicare/physician-fee-schedule/search?Y=0&T=4&HT=0&CT=0&H1=G0108&M=5

6
https://www.cms.gov/medicare/physician-fee-schedule/search?Y=0&T=4&HT=0&CT=0&H1=95251&M=5

7
https://www.salary.com/research/salary/benchmark/diabetes-educator-salary

8
https://www.cms.gov/medicare/physician-fee-schedule/search?Y=0&T=4&HT=0&CT=0&H1=95251&M=5

## Abbreviations

Remote patient monitoring (RPM); Type 1 diabetes (T1D); Continuous glucose monitoring (CGM); Certified diabetes care and education specialist (CDCES); Lucile Packard Children’s Hospital Stanford (LPCH); Teamwork, Targets, Technology and Tight Control (4T); Timely Interventions for Diabetes Excellence (TIDE); Fee-for-Service (FFS).
